# Nutrient Sensing via mTOR in T Cells Maintains a Tolerogenic Microenvironment

**DOI:** 10.3389/fimmu.2014.00409

**Published:** 2014-08-28

**Authors:** Duncan Howie, Herman Waldmann, Stephen Cobbold

**Affiliations:** ^1^Sir William Dunn School of Pathology, University of Oxford, Oxford, UK

**Keywords:** mTOR, metabolism, immune regulation, T cell differentiation, tolerance

## Abstract

We have proposed that tolerance can be maintained through the induction, by Treg cells, of a tolerogenic microenvironment within tolerated tissues that inhibits effector cell activity but which supports the generation of further Treg cells by “infectious tolerance.” Two important components of this tolerogenic microenvironment depend on metabolism and nutrient sensing. The first is due to the up-regulation of multiple enzymes that consume essential amino acids, which are sensed in naïve T cells primarily via inhibition of the mechanistic target of rapamycin (mTOR) pathway, which in turn encourages their further differentiation into FOXP3^+^ Treg cells. The second mechanism is the metabolism of extracellular ATP to adenosine by the ectoenzymes CD39 and CD73. These two enzymes are constitutively co-expressed on Treg cells, but can also be induced on a wide variety of cell types by TGFβ and the adenosine generated can be shown to be a potent inhibitor of T cell proliferation. This review will focus on mechanisms of nutrient sensing in T cells, how these are integrated with TCR and cytokine signals via the mTOR pathway, and what impact this has on intracellular metabolism and subsequently the control of differentiation into different effector or regulatory T cell subsets.

## Introduction

The mechanistic target of rapamycin (mTOR) signaling acts as a principle integrator of nutrient-sensing pathways that control and coordinate the metabolism of the cell according to its need to proliferate or functionally differentiate (1, 2). When a naïve or resting T cell recognizes its cognate antigen, the activation process involves synthesis of many new proteins, the induction of rapid cell proliferation, cytokine driven differentiation toward a range of effector functions, and chemokine induced cell movement to any site of inflammation. All these processes require a rapid increase in the main source of energy for the cell, which is ATP. While oxidative phosphorylation (OXPHOS) by the mitochondria is the most efficient means to generate large amounts of ATP, there seems to be a switch from primarily OXPHOS in resting T cells to an aerobic form of glycolysis, known as the “Warburg effect” (3), during activation and proliferation (4). This may be because glycolysis can use glucose as the basic source of carbon to generate many of the fundamental building blocks of the proliferating cell, such as amino acids, lipids, complex carbohydrates, and ribonucleotides (5). The mTOR pathway is strongly implicated in this metabolic switch because its activation up-regulates the surface expression of the glucose transporter, Glut1, probably as a result of TCR and CD28 signaling through phosphatidylinositide 3-kinase (PI3K) and protein kinase B (PKB also known as AKT) (6). AKT signaling via mTOR also leads to higher expression of amino acid and other nutrient transporters, such as the transferrin receptor (7). Signaling downstream of mTOR via ribosomal S6 kinase and 4E-BP1 is also required to initiate protein synthesis from mRNA at the ribosome (8). Rapamycin is a drug (trade name sirolimus) that inhibits mTOR by forming a complex with FKBP12, which binds to raptor and disrupts the activity of the mTORC1 complex. Rapamycin is used clinically as an immunosuppressive agent, particularly in allogeneic transplantation, and has over recent years gained interest as a potential alternative to calcineurin inhibitors, which not only have renal toxicity but are also thought to block the induction of regulatory T cells (9).

## Mechanisms of Peripheral Tolerance

### Regulatory T cells are enriched within tolerated tissues

It has recently become clear that tolerance is associated with Treg cells that act within a highly localized microenvironment to maintain a state of acquired immune privilege (10, 11). Tolerance to skin grafts can be induced using a short course of non-depleting CD4 antibodies in mice expressing a TRC transgenic, monoclonal population of CD4^+^ T cells such that every T cell recognizes the male antigen presented by MHC-II on the graft (12). This tolerance is not due to clonal deletion as the graft recipients contain normal numbers of male specific T cells, including a proportion that show evidence of recent activation, by expression of CD44 and IL-2. As these mice are on a RAG^−/−^ background, there are no FOXP3^+^ Treg cells present in the naïve animal pre-grafting, but after tolerance induction, peripherally induced, FOXP3^+^ Treg cells are found gradually increasing over time (up to 50%) within the tolerated graft tissue, but only in small numbers (1–2%) in the lymph nodes or spleen (12). This suggests that the Treg cells are acting to control the response of effector T cells primarily within the graft itself.

**Figure 1:**
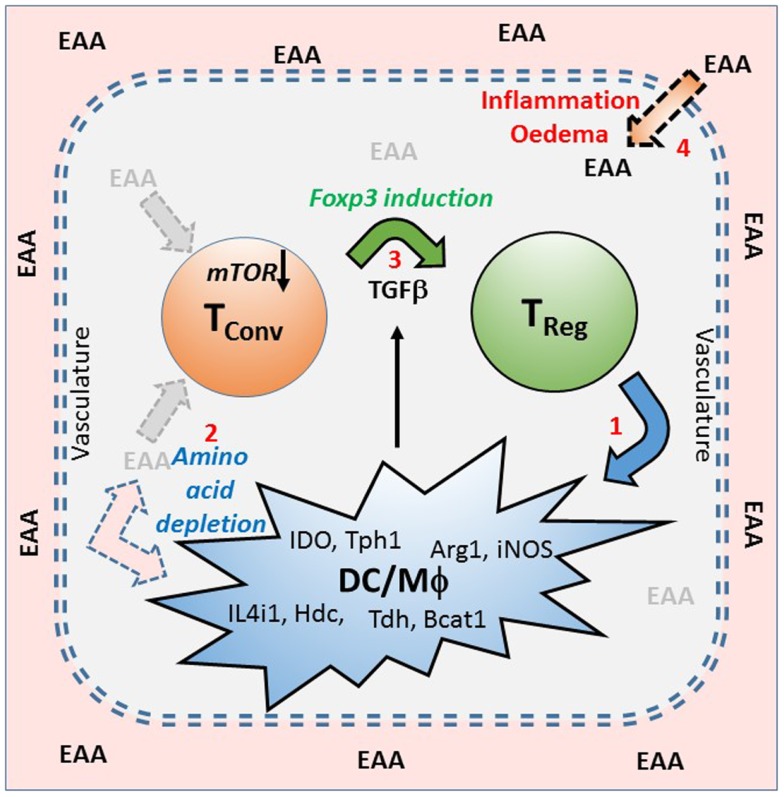
**A model of infectious tolerance that depends on a nutrient depleted microenvironment maintained by Treg cells within tissues**. This model proposes that immunological tolerance is maintained within tissues by the localized depletion of nutrients, particularly the essential amino acids (EAA), which are required for the proliferation and effector function of conventional T cells (Tconv). Amino acid depletion is primarily as a result of regulatory T cells (Treg) inducing (1), in dendritic cells (DC) and macrophages (Mϕ), a range of enzymes that catabolize (2) or utilize EAA (examples are shown). This lack of EAA is sensed via the mTOR pathway which, in the presence of TGFβ, encourages the expression of FOXP3, and the induction of further Treg (3). Under conditions of tolerance the intact vasculature maintains a barrier between the blood and the tissues, but if there is inflammation or damage to the vasculature, causing edema, then EAA can leak into the tissues (4) and contribute to a breaking of the tolerant microenvironment.

This can be demonstrated where alloantigen specific tolerance has been induced to a skin graft (e.g., by a short period of coreceptor blockade with non-depleting anti-CD4 and CD8 monoclonal antibodies), and then that tolerated graft has been removed and re-transplanted onto a secondary recipient with no immune system of its own (e.g., a recombinase activating gene 1 knock out mouse). This skin graft is accepted by the secondary recipient as it has no T cells to cause any rejection. If, however, we treat the recipient at the time of graft transfer with monoclonal antibodies that inactivate or deplete FOXP3^+^ Treg cells (e.g., anti-CD25, or if the original recipient carries the hCD2.FOXP3 knock in reporter, anti-hCD2), the transferred skin grafts are rapidly rejected (11, 13). This shows that the re-transplanted, originally tolerated skin graft carried over within it perfectly functional effector T cells, but that it also contained FOXP3^+^ Treg cells that were actively blocking the ability to cause rejection. By studying the changes in gene expression between tolerated and rejecting skin grafts, and comparing dendritic cells (DCs) when they interact with Treg cells in the presence or absence of antigen (14–16), it was found that while co-stimulatory ligands and antigen presentation by DCs were down-regulated, there was also an up-regulation of a number of enzymes that either catabolize or utilize essential amino acids (EAAs) (17). In the context of a restricted microenvironment within tissues, where there may not be free exchange of amino acids and other nutrients with plasma in the vasculature, the local depletion of EAAs by these enzymes could be an effective mechanism to control the immune response via the mTOR nutrient-sensing pathway (Figure 1). Conversely, edema and breakdown of the vasculature may provide an excess of amino acids that would promote T cell activation and graft rejection. This regulation by amino acid availability might be particularly effective if regulatory T cells were more resistant to the effects of amino acid starvation. It has been shown that the intracellular concentration of leucine, a particularly strong activator of mTOR, is controlled by a TCR induced expression of the neutral amino acid transporter slc7a5 in Th1 and Th2 effector T cells, where it is required for their activation and differentiation, but regulatory T cells seem not to depend on this particular transporter (18).

### IDO mediated tryptophan catabolism as a mechanism of immune regulation

The maternal immune response to paternal alloantigens expressed on the developing fetus is in many ways similar to that seen in transplantation. The expression of the enzyme indoleamine 2,3 dioxygenase (IDO) in the placenta during pregnancy was shown to be important for avoiding that immune response by the finding that a specific inhibitor, 1-methyl tryptophan, could induce spontaneous abortion of semi-allogeneic, but not syngeneic, conception (19). *In vitro* experiments showed that IDO seemed to act primarily through depletion of tryptophan, although there is some evidence that the kynurenine products of tryptophan catabolism may also play a role (20). The tryptophan depletion is sensed, at least in part, by general control non-repressed 2 (GCN2), which is one of the initiators of the integrated stress response, activation of which leads to a block in the proliferation of CD8 effector T cells (21). GCN2 is also required for the survival of T cells, including CD4^+^ Treg cells, during periods of amino acid starvation (17), but it was not essential for T cells to sense the absence of other EAAs and halt their proliferation *in vitro* (17). The *in vitro* induction of forkhead box P3 (FOXP3) as a result of stimulating naïve CD4^+^ T cells in the presence of low doses of TGFβ was also unaffected by activating the GCN2 pathway with histidinol (an inhibitor of histidyl-tRNA synthetase) while in contrast, inhibition of the mTOR pathway with rapamycin gave a synergistic increase in FOXP3 expression (17). It has recently been found that tryptophan levels can be sensed via mTOR and PKCθ signaling (22).

### Depletion of essential amino acids maintain an immune privileged microenvironment within tolerated tissues

Indoleamine 2,3 dioxygenase may have been the first example of immune regulation due to amino acid catabolism because tryptophan is thought to be present at the lowest concentration of all the EAAs, at least in the plasma. Recently, it has been shown that mast cells that seem to be specifically associated with tolerated skin grafts, express the enzyme tryptophan hydroxylase (TPH1) (23), which utilizes tryptophan to synthesize serotonin. TPH1 knockout mice, unlike wild type controls, could not be made tolerant of allogeneic heart grafts using costimulation blockade, but this could be reconstituted with wild type mast cells. Providing 5-hydroxytryptophan to bypass the defect in serotonin synthesis in TPH1 knockout mice was not sufficient to allow the induction of tolerance, suggesting that the mechanism was dependent on tryptophan depletion rather than serotonin synthesis (24). Similarly, arginase (ARG1) expression has been implicated in regulating the immune response during pregnancy (25, 26) and has also been associated with a presumed protective, type 2, population of macrophages within tissues (27). Arginine is the substrate for the inducible form of nitric oxide synthase (iNOS), which is normally associated with classically activated macrophages and a Th1 effector cell response, but under limiting concentrations of arginine *in vitro*, both arginase and iNOS can cause sufficient depletion of arginine to cause mTOR inhibition and subsequently block T cell proliferation (17). Another enzyme called IL4-induced 1 (IL4i1) for its induction in myeloid cells under Th2 conditions, depletes EAAs with hydrophobic side chains such as phenylalanine (28). IL4i1 was also found to be induced in DC when co-cultured with Treg cells (17).

Expression of many of these EAA consuming enzymes could be induced within skin grafts *in vivo* (17) and in DCs *in vitro* (17) by a cognate interaction with antigen specific Treg cells, either by specific cytokines such as TGFβ, IL4, or interferon-γ (IFN-γ) or via cell surface interactions such as CTLA4 (17). In addition, catabolic enzymes specific for threonine (threonine dehydrogenase – TDH) and the branched chain amino acids (branched chain amino acid aminotransferase – Bcat1) were more closely associated with the inflammation and wound healing even when skin was grafted onto recipients with no adaptive immune system (17). This suggests that tissues such as skin have a built in nutrient-sensing mechanism for protecting themselves against immune attack that might be important for maintaining self-tolerance, which might explain why long-term surviving, fully healed in syngeneic skin grafts also had higher levels of these particular enzymes, as well as an increased infiltration by FOXP3^+^ Treg cells (16).

All these observations led us to propose that tolerance may be maintained by regulatory T cells that induce a tolerogenic microenvironment within tissues that is, at least in part, dependent on the induction of many different enzymes that deplete the local pool of EAAs. This lack of EAAs is sensed by T cells via the mTOR pathway, which inhibits the generation and function of effector T cells, while encouraging the development of further FOXP3^+^ Treg cells (Figure 1). This mechanism may explain the phenomenon known as “infectious tolerance” where it was shown that naïve T cells that co-existed with regulatory T cells in a tolerant environment acquired all the properties of the original tolerant T cells within 3 weeks, such that tolerance was maintained if the original cohort of tolerant T cells were subsequently depleted (29). The question then arises as to how the consequent inhibition of mTOR regulates the activation and differentiation of different functional T cell subsets.

## mTOR Integrates Nutrient Sensing and Activation Signals in T Cells

### The mTOR pathway in T cells

**Figure 2:**
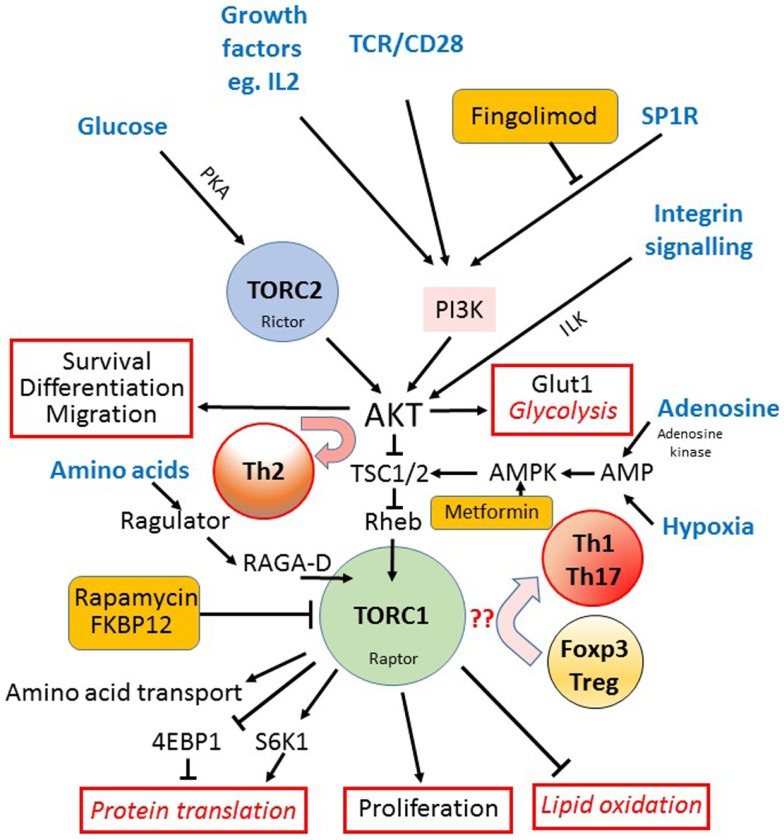
**The mTOR pathway in T cells**. The mechanistic target of rapamycin (mTOR) is a component of both the TORC1 and TORC2 signaling complexes. The TORC1 complex acts as the main integrator of many different signals (input signals shown in blue text) from nutrients such as glucose, via TORC2, the TCR, costimulation and growth factors, via PI3K and AKT, and amino acids via the regulator complex. Hypoxia and AMP levels are also sensed via AMPK and TSC1/2. AKT activation downstream of TORC2 is important for cell survival, drives the expression of the glucose receptor (Glut1) and glycolytic metabolism, and is required for the differentiation into Th2 cells (outputs of signaling shown outlined in red). TORC1 is important for the initiation of mRNA translation via S6K1 mediated phosphorylation of the ribosomal protein S6, and the up-regulation of amino acid transporters at the cell surface. TORC1 also activates lipid oxidation and cell proliferation while it inhibits the expression of FOXP3 and Treg differentiation in favor of Th1 and Th17 cells. The sites where three different clinically available drugs (rapamycin, fingolimod, and metformin) impact on the mTOR pathway are indicated (orange boxes).

The mTOR pathway (Figure 2) acts generally to coordinate many aspects of cell growth and metabolism, including the response to hypoxia and the biogenesis and oxidative capacity of mitochondria (30). mTOR forms two distinct complexes that seem to have different signaling functions (TORC1 and TORC2) (31). TORC1 is thought to be the main nutrient-sensing complex and is composed of the serine/threonine kinase mTOR itself, the scaffolding protein raptor, the positive accessory proteins FKB12, deptor, and mLST8, and a regulatory subunit PRAS40 that is a target of AKT downstream of PI3K signaling (32). Most signals, which eventually lead to activation of the TORC1 complex, including glucose, cytokines, growth factors, and costimulation in T cells, do so via PI3K signaling, which eventually phosphorylates mTORC1 via the tuberous sclerosis (TSC) 1/2 complex and the ras homolog expressed in brain (Rheb). Rheb is localized within the cell in a Rab7^+^ lysosomal compartment and the interaction between TORC1 and Rheb is entirely dependent on the sensing of sufficient amino acids. Although the molecular sensor of amino acids in mammals remains unclear, downstream signaling requires the four ras-related GTP binding (or RAG GTPase – RRAG) proteins (A–D) together with the ragulator complex (33, 34), so that a lack of available amino acids acts as a potent inhibitor of TORC1 activity. The immunosuppressive drug rapamycin binds to FKB12 and disrupts the formation and function of the TORC1 complex (35) and therefore has a similar effect on cells as does amino acid starvation. Conversely, TORC1 activation drives protein synthesis via phosphorylation of S6K1, which phosphorylates the ribosomal protein S6 and initiates the translation of messenger RNA. At the same time, 4E-BP1, an inhibitor of protein translation, is also deactivated by mTOR-mediated phosphorylation.

Much less is known about how the TORC2 complex is regulated: there is some evidence that it senses reactive oxygen species and is involved in sphingolipid homeostasis at the plasma membrane (36), while it also seems to sense glucose availability via a cAMP/PKA pathway (37). TORC2 is thought to be negatively regulated by TORC1 activity via Sin1 phosphorylation (38). Rapamycin therefore indirectly activates TORC2 in the short term, but chronic long-term inhibition (over hours to days) of TORC1 (39) or by amino acid starvation (40) seems to eventually reduce the activity of TORC2. TORC2 controls various spatial aspects of cell growth, in particular cell polarity and responses to chemotactic signals via G protein coupled activation of RAS (41).

### mTOR signaling inhibits FOXP3 expression

It has long been known that mTOR inhibition by rapamycin is potently immunosuppressive, partly because it blocks the ability of T cells to respond to interleukin 2 (IL-2) signaling via PI3K and consequently their ability to proliferate in response to antigen (42). More recently, it is has become clear that mTOR signaling also controls the differentiation of CD4^+^ T helper cell subsets (43), and in particular, the expression of the “master” transcription factor for regulatory T cells, FOXP3. mTOR activation downstream of the TCR, CD28 costimulation and cytokine mediated PI3K signaling is generally required for the proliferation and differentiation of effector T cells but this is inhibitory for FOXP3 expression (44, 45). Signaling downstream of the sphingomyelin phosphate receptor (S1PR), which is required for lymphocyte trafficking and exit from the lymph nodes, can also activate mTOR (46). Interestingly, this pathway is the target of the immunosuppressive drug known as Fingolimod/FTY720 (47), which also has the potential to promote Treg cell development (48). Although the exact mechanism by which mTOR inhibition enhances FOXP3 expression has not been clarified, there is some evidence that implicates a number of different pathways. These could act via poorly defined effects on FOXP3 translation via inhibition of S6K1 and reduced phosphorylation of the ribosomal protein S6. Additionally, mTOR could act either indirectly via suppressor of cytokine signaling 3 (SOCS3) (49, 50) or directly on signal transducer and activator of transcription 3 (STAT3) downstream of IL-6 and the satiety hormone leptin (51). Phospho-STAT3 may then compete for the IL-2 driven STAT5 enhancement of FOXP3 transcription (52). In addition, FOXO3a (53, 54) and the TGFβ signaling component SMAD3, two transcription factors promoting FOXP3 expression, are negatively regulated by AKT downstream of TORC2 (55). Evidence from mice with T cell targeted deficiencies in either raptor (TORC1) or rictor (TORC2) suggests that TORC1 tends to promote Th1 differentiation (43) while TORC2 may bias toward Th2 via AKT and PKCθ (56). Inhibition of both complexes seems to be required for the optimal induction of FOXP3^+^ Treg cells while Th17 cell development seems to be independent of TORC2, but is inhibited by rapamycin in favor of FOXP3^+^ Treg cells (57).

### While mTOR inhibition is required for FOXP3 expression, mTOR activation is needed for regulatory function

Mechanistic target of rapamycin inhibition therefore seems to be associated with tolerance and FOXP3^+^ Treg cell induction, and this appeared to be confirmed by T cell specific mTOR knockout mice, which develop an excess of FOXP3^+^ Treg cells over Th1 and Th2 effector cells (43). Recent data, however, from FOXP3-Cre.Raptor^fl/fl^ mice where TORC1 activity has been specifically knocked out in FOXP3^+^ Treg cells, indicates that TORC1 activation is still required for Treg cells to function, as evidenced by the development of an autoinflammatory condition very similar to scurfy or FOXP3 deficient mice (58). CD4–Cre.Raptor^fl/fl^ mice, lacking TORC1 activity in all T cells, however, did not develop disease, presumably because this also compromised the effector T cells. This raises the possibility that the optimal induction and expansion of FOXP3^+^ Treg cells takes place in the nutrient depleted microenvironments associated with tolerance, but the Treg cells only become fully active and proliferative when there is inflammation that needs to be controlled, which requires a re-activation of their mTOR pathway. Interestingly, it had previously been postulated that the optimal functional induction of FOXP3^+^ Treg cells required alternate cycles or oscillations of mTOR inhibition, first to promote induction, and subsequently mTOR activation to promote proliferation (59).

### Modulation of FOXP3 expression by adenosine and hypoxia

Hypoxia induced factor (HIF) 1α, another downstream target of TORC1, has also been implicated either as a positive (60, 61) or a negative (62, 63) regulator of FOXP3 expression. HIF1α is a BHLH-Pas transcription factor that has an essential role in the response of cells to hypoxia and which is able to bind directly to FOXP3 protein to target it for proteosomal degradation (62). The level of HIF1α transcription is controlled by NFκβ (64), but its activity is mainly controlled post-translation by an oxygen mediated ubiquitination and degradation controlled by the von Hippel-Lindau tumor suppressor complex with additional positive regulation via a TORC1 mediated phosphorylation (65). Activation of naïve T cells under hypoxic conditions has also been suggested to enhance FOXP3 expression and the differentiation to Treg cells (60), but it is not clear whether this is a direct effect of HIF1α on FOXP3 expression, or whether it is an indirect effect of HIF1α feedback inhibition of mTOR (66). Hypoxia is associated with raised levels of AMP within the cell, and the enzyme AMP activated protein kinase (AMPK) causes inhibition of mTOR via TSC1/2 (67, 68).

**Figure 3:**
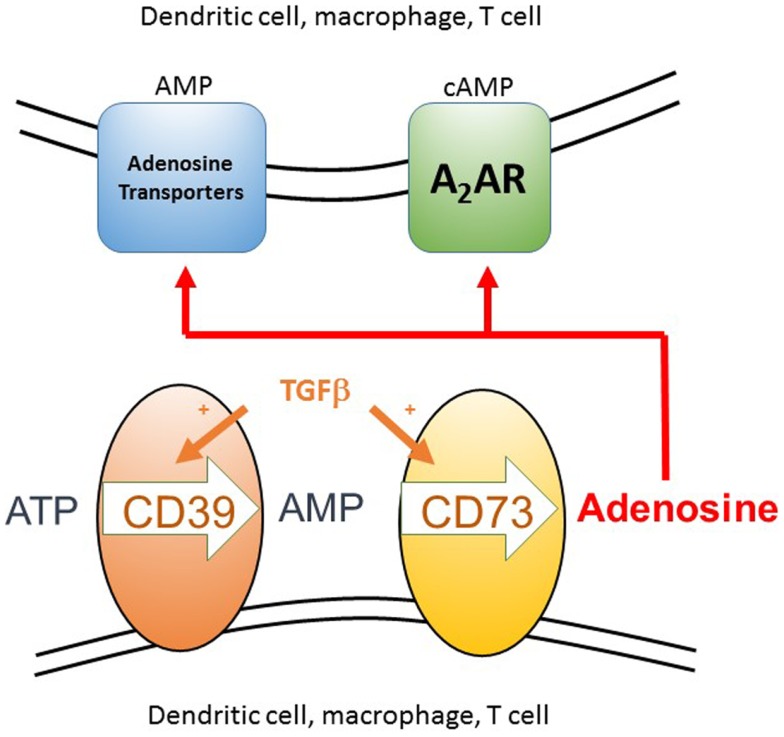
**The generation of extracellular adenosine as a component of an anti-inflammatory microenvironment**. Extracellular ATP arises as a result of cell death, either from the host or pathogen, as can itself be inflammatory. The two ectoenzymes CD39 and CD73 are normally co-expressed constitutively on Treg cells, but can be induced on the surface of many different cell types, including conventional T cells, dendritic cells, and macrophages, in the presence of a source of TGFβ. These enzymes sequentially convert extracellular ATP to AMP and then adenosine. Adenosine can then act either by binding to the A2A receptor on T cells and DC, which signals via cAMP or it can be taken up via adenosine transporters where it is rapidly converted to intracellular AMP by adenosine kinase. These two signaling pathways act to inhibit inflammation and T cell proliferation. AMP activated kinase mediated inhibition of the mTOR pathway can then occur downstream of either signaling pathway.

AMP and adenosine are particularly relevant to immune regulation, as TGFβ is able to induce in a range of hematopoietic cells the co-expression of two ectoenzymes, CD39 and CD73 (69) that are also constitutively expressed on Treg cells (70). These two enzymes (Figure 3) act at the cell surface to convert extracellular sources of ATP, which is associated with inflammation and cell necrosis, into the anti-inflammatory product adenosine. Extracellular adenosine can generate the second messenger cAMP within the target cell via activation of specific G protein coupled receptors on the cell surface [e.g., A_2A_R on T cells (71, 72)] or it can be directly taken up by specific adenosine transporters (73) where, once inside the cell, it is rapidly converted to AMP by adenosine kinase. AMP is also generated in the cell downstream of G protein signaling via cAMP, which is subsequently broken down to AMP by phosphodiesterases. Although there is evidence that this pathway is relevant to tumors escaping immune surveillance (74, 75), it remains, however, to be resolved whether adenosine is as an important component of the anti-inflammatory microenvironment within tolerated tissues.

## T Cells Display Metabolic and Functional Plasticity in Response to Diverse Environmental Cues

**Figure 4:**
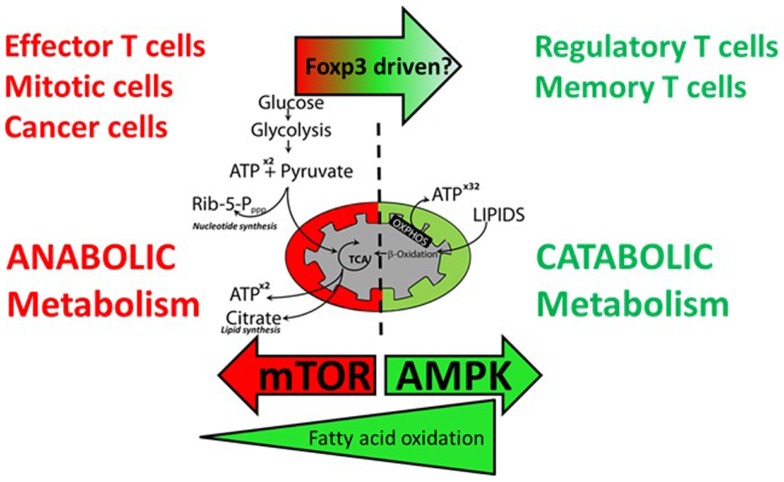
**Effector T cells and regulatory T cells have different requirements for anabolic and catabolic metabolism**. Effector T cells, proliferating T cells, and cancer cells favor anabolic metabolism using aerobic glycolysis to fuel energy and cell mass demands during proliferation. Activation of mTOR drives this metabolic shift. Precursors for nucleotide synthesis and lipid synthesis are supplied via the pentose phosphate pathway and the TCA cycle, respectively. Resting regulatory and memory T cells do not require glycolysis and TCA intermediates for cell growth and favor catabolic metabolism fueled by β-oxidation of fatty acids to fuel oxidative phosphorylation. Inhibition of mTOR and activation of AMPK favor a shift to catabolic metabolism.

T cells not only adapt to their environment by changing metabolic mode, but in addition their chosen fuel source and metabolites, to a large extent, affect their fate and function (Figure 4). T cells use glucose and glutamine as their primary source of energy but can switch to ketone bodies and fatty acid use under certain circumstances (76). Glucose is the primary substrate for ATP production in T cells (77, 78). During glycolysis, glucose is converted to two molecules of pyruvate and two molecules of ATP in an oxygen-independent process. Pyruvate generated from glycolysis is oxidized in the TCA cycle yielding NADH and FADH2, which is used to fuel OXPHOS. OXPHOS is oxygen dependent and efficiently yields as much as 36 molecules of ATP per molecule of glucose. In order to mount an effective immune response T cells must expand rapidly and can achieve doubling times as low as 2–6 h (79). To fuel this expansion T cells undergo a major metabolic shift from primarily catabolic fatty acid oxidation (FAO) driven OXPHOS to anabolic glycolysis and glutaminolysis during activation, then revert back to FAO in the memory phase. Glycolysis is an amphibolic process that although less efficient in net ATP production, fuels rapid T cell growth by providing NADPH and ribose from the pentose phosphate pathway for reductive biosynthetic reactions and nucleotide synthesis and fuels lipid synthesis via citrate from the TCA cycle. During this process, glucose is incompletely oxidized and is fermented to lactate while glutamine is converted to glutamate, aspartate, and ammonia. This shift to oxygen-independent glycolysis in the context of normoxia has been termed aerobic glycolysis and is a feature of cancer cells, where the process is termed Warburg metabolism, reviewed in Ref. (80).

Multiple environmental nutritional signals are integrated by T cells via mTOR and AMPK to control their choice of metabolism and function. These signals include glucose and glutamine concentration, oxygen tension, amino acid concentration, lipids, salt concentration (NaCl), leptin concentration, and ATP:ADP ratio. In addition, immune-specific inputs such as T cell receptor triggering and co-stimulatory/inhibitory signals and cytokines are integrated by T cells to change their metabolic mode.

### Glucose is required for T cell activation

Glucose is critical for T cell activation. It is the primary carbon source for macromolecules such as lipids and nucleotides in T cells and can fuel the pentose phosphate pathway to generate NADPH reducing equivalents. During activation the T cell increases its rate of glucose metabolism and up-regulates cell surface Glut1 receptors to transfer glucose from the extracellular space (81, 82). Concomitant with the increase in surface Glut1, hexokinase is also upregulated (83, 84) after T cell activation. Hexokinase phosphorylates glucose thus trapping it in the cytoplasm and maintaining a glucose gradient across the plasma membrane to maintain facilitated diffusion of glucose. Indeed, even in the presence of glutamine, in the absence of glucose T cell survival and proliferation is severely impaired (77). Effector T cells (Teff) specifically Th17, Th1, and Th2 are particularly glycolytic and dependent on glucose and accumulate preferentially in Glut1 transgenic mice at the expense of Treg cells. Treg cells preferentially use fatty acids as a fuel, their development is enhanced in the presence of excess fatty acids (78).

### Glutamine as an important carbon source for T cells

Glutamine is the most abundant amino acid in serum (85) and has also been implicated in immune regulation (86). It is essential for T cell activation as a source of nitrogen and as a key anapleurotic substrate enabling nucleotide synthesis and redox control in addition to fueling metabolism via the TCA cycle following conversion to α-ketoglutarate. T cells consume glutamine at an equivalent rate to glucose (87, 88). Activation of T cells triggers a rapid 5- to 10-fold increase in SNAT1 and SNAT2 (89) (sodium dependent neutral amino acid transporter) glutamine transporter expression and glutamine import via a CD28 and ERK/mitogen-activated protein kinase (MAPK) dependent mechanism. Naïve T cells transport glutamine into the cell via ASCT2 (slc1a5) (90) where the concentration of glutamine becomes sufficient to act as an efflux substrate to drive the system L neutral amino acid transporter slc7a5 in complex with CD98 (slc3a2) to import neutral amino acids into the cell. Sustained neutral amino acids and glutamine are essential for TCR/CD28 driven mTORC1 activation, but not other TCR signaling pathways such as MAPK or IKK (90). Activated TORC1 subsequently prolongs glutamine anapleurosis by activating glutamate dehydrogenase via indirectly inhibiting transcription of its inhibitor, SIRT4 (91). Glutamine can fuel the TCA cycle for anabolic and catabolic metabolism in the presence or absence of glucose and in the presence of hypoxia (92). Cells require acetyl coenzyme A for lipid synthesis. During hypoxia or active proliferation where aerobic glycolysis is engaged glucose carbons are converted to lactate and diverted away from the TCA cycle. Under these conditions cells can use reductive metabolism of α-ketoglutarate as an alternative anaplerotic route to produce acetyl co-A for the synthesis of lipids (93).

### Fatty acid metabolism

Resting naïve T cells, memory CD8 T cells and resting regulatory T cells share FAO as a common default metabolic mode (78, 94–96). This metabolic state enhances ATP production through mitochondrial OXPHOS while minimizing anabolic processes required for increased cell mass during proliferation. Environmental lipid concentration has a role in determining the fate of differentiating T cells. Treg cell homeostasis requires environmental lipids, which activate the nuclear receptors peroxisome proliferator activated receptor (PPAR)α and PPARγ that function as fatty acid sensors and regulators of lipid metabolism. These receptors promote FOXP3 expression by CD4 T cells in response to TGFβ (97). Clinically, PPARγ agonists downregulate the production of pro-inflammatory cytokines such as IL-6, TNFα, and leptin (98). Provision of fatty acids to T cells *in vitro* promotes differentiation to Treg cells while inhibiting effector differentiation (78). These observations may explain the severe acute immunosuppression associated with calorific starvation observed in humans.

### Dietary NaCl and inflammation

Recent evidence suggests that dietary sodium chloride concentration may play a role in controlling inflammation by inhibiting induction of peripheral Treg cells and favoring the induction of Th17 and Th2 cells (99, 100). Elevated levels of NaCl by just 40 mM have been shown to activate p38 MAPK signaling, resulting in activation of the osmosensitive form of NFAT5 (also known as TONEBP) leading to activation of serum glucocorticoid kinase 1 (SGK1), an AGC serine/threonine kinase (99). SGK1 has been shown to govern salt transport and salt homeostasis in multiple cell types dependent on TORC2 activity (101). Raised levels of salt were shown to turn on SGK1 expression, enhance IL-23R expression, and augment TH17 cell differentiation (99, 100). Powell and colleagues showed that after activation by mTORC2, SGK1 promoted T helper type 2 (T_H_2) differentiation by negatively regulating degradation of the transcription factor JunB mediated by the E3 ligase Nedd4-2 (102). The same group also showed that SGK1 turns off IFN-γ via TCF-1. Sodium chloride concentrations vary anatomically, the concentration in plasma is approximately 140 mM, whereas in insterstitium and lymph nodes it is much higher ranging from 160 to 250 mM (103, 104). Thus, it is possible that sodium concentration limits pro-inflammatory activation of T cells in the blood while allowing Th17 differentiation in tissues and lymph nodes.

### Leptin as a pro-inflammatory cytokine

Leptin is an IL-6-like cytokine produced by adipocytes (termed an “adipokine”), which acts directly on the hypothalamus as a satiety hormone and also has effects on metabolism and T cell functions (105, 106). Adipokines are hormones or cytokines secreted by adipocytes, which have pleiotropic effects on the nutritional status and immune system of the organism. These include the cytokines IL-1, IL-6, IFN-γ, TNFα as well as leptin and adiponectin. Leptin is produced at high levels constitutively by regulatory T cells, which also express the leptin receptor (ObR) (107). Leptin is required for activated T cell proliferation and cytokine production in part via inducing up-regulation of mRNA and surface expression of the Glut1 receptor and glucose uptake (108). Leptin in combination with T cell receptor triggering induces activation of CD4^+^CD45RA naïve T cells but inhibits activation of CD4^+^CD45RO^+^ memory T cells in humans (109). It skews these cells to produce pro-inflammatory cytokines including IFN-γ and TNFα and leptin itself. Leptin functions to negatively regulate Treg cell activity and proliferation. Leptin deficient mice (*ob/ob*) and leptin receptor deficient mice (*db/db*) have decreased susceptibility to autoimmunity and increased numbers of Treg cells (107, 110, 111). Neutralization of leptin in Treg cell cultures enhances their IL-2 dependent proliferation while maintaining suppressive function. Thus, leptin appears to function as a feedback control mechanism to control Treg cell activity in response to nutrient availability.

## Signaling Mechanisms Regulating T Cell Metabolism

### mTOR coordinates metabolism and T cell differentiation

The activation of naïve T cells has been primarily associated with glucose metabolism, even under aerobic conditions, as this not only provides a source of ATP for energy and effector cell activity but also generates the precursors for nucleotide synthesis and lipogenesis that are required for cell proliferation (5). Under conditions of nutrient restriction and mTOR inhibition, however, it would be expected that T cells would switch to more efficient pathways of energy generation, such as OXPHOS and FAO, both of which require active mitochondria. Indeed, it has been shown that Treg cells have higher levels of AMPK activity, which as we have seen leads to mTOR inhibition, and this in turn reduces the expression of the glucose transporter (Glut1) and enhances lipid oxidation, effects, which can be reversed in Glut1 over-expressing transgenic mice (78). Multiple intracellular signaling pathways control the choice of metabolic activity engaged by T cells.

### c-Myc

The proto-oncogene c-Myc is a critical positive regulator of both gylcolysis and glutaminolysis and as such has a potentially important role in T cell plasticity. c-Myc and its binding partner max binds to over 10,000 sites in mammalian genomes at a consensus E-box sequence CAGCTG (112, 113). c-Myc has a fundamental role in controlling metabolism. It increases transcription of all the glycolysis genes (114) and also the glutamine transporters ASCT2 and SN2 by binding to their promoters (114–116). c-Myc increases usage of pentose phosphate pathway, glycolysis, and glutaminolysis (84) and also augments mitochondrial biogenesis via up-regulation of PGC1 (117–119) and the transferrin receptor TFRC (120), which is necessary to provide iron for the heme containing proteins of the electron transport chain. Thus, c-Myc drives cells toward anabolic metabolism, at the same time it promotes cell division via glutaminolysis providing the anaplerotic substrate aKG needed for synthesis of polyamines required for T cell proliferation (115). Myc deletion in T cells using inducible tamoxifen cre-lox systems leads to inhibition of glycolysis and glutaminolysis (84). Glutamine deprivation inhibits T cell activation induced growth and proliferation (84).

### Estrogen receptor related receptor α

Estrogen receptor related receptor α (ERRα) is an orphan receptor, one of three members of a family α, β, and γ, which bind to a DNA consensus site termed the ERR response element (TNAAGGTCA) in multiple genes (121). Despite its name it is not activated by estrogen or related hormones, but seems to be constitutively active, having an active ligand binding region in the absence of ligand (122). ERRα is important in immune reprograming as it seems to function to “rewire” cells to use glucose for anabolism (123). The glucose transporter glut1 and glucose uptake are inhibited in ERRα null T cells, and by chemical inhibition of ERR (123). ERRα physically interacts with PGC1α and PGC1β (124), which act as co-activators to activate transcription of a number of genes important for FAO (MCAD, CPT1B), TCA cycle (IDH3A, AC02), and OXPHOS (CYCS, ATP5B) in multiple cell types (125). Interaction of ERRα with the transcriptional co-repressor RIP140 results in down regulation of many of the genes, which PGC1α/β activates (126–128). Acute inhibition of ERRα in T cells results in their inability to proliferate or differentiate into Th subsets, an effect, which is rescued for proliferation and Treg cell differentiation, but not Teffector differentiation, by addition of long chain fatty acids (123). Thus, ERRα functions in T cells to enable them to prepare for the metabolic demands of proliferation and differentiation into effector subsets by enhancing glucose uptake and mitochondrial biogenesis.

### PGC1α

Peroxisome proliferator activated receptor γ co-activator 1α (PGC1α) is a transcriptional co-activator, a protein with ability to enhance transcription factor binding to genes, which has no specific DNA binding capability of its own. It has a central role in metabolism being the co-activator for multiple transcription factors involved in mitochondrial biogenesis (129) and glucose and fatty acid metabolism (130) and gluconeogenesis (131).

### Liver X receptor

Liver X receptors (LXRs) are receptors of the nuclear receptor family, which bind to endogenous oxysterols. LXRs have two isoforms, α and β. Both isoforms are expressed by CD4 T cells. These receptors heterodimerize with the retinoid X receptor (RXR) and function to modulate cholesterol homeostasis by controlling genes involved in cholesterol and lipid metabolism including sterol regulatory element binding protein (SREBP) (132, 133). LXRs have potent effects on T cell function including inhibition of lymphocyte proliferation (132). Ectopic expression of LXR was also shown to inhibit T cell differentiation into Th17 cells via induction of srebp-1 a protein capable of inhibition of aryl hydrocarbon receptor (Ahr) binding to the IL-17 gene (134). Agonists of LXR have been shown to ameliorate experimental autoimmune encephalomyelitis (EAE) (134). SREBP-1 has recently been shown to be essential for coordinating T cell receptor activation and lipid anabolism in dividing CD8 T cells (135). In the absence of SREBP-1, CD8 T cells can enter G1 phase of cell cycle but fail to continue to mitosis due to a lack of sufficient cellular cholesterol.

### Ca^2+^ signaling

Calcium levels in the mitochondrial matrix are tightly regulated by mitochondrial Ca^2+^ uniporters (MCU), which transport Ca^2+^ across the inner mitochondrial membranes (136). Mitochondrial sequestration of Ca^2+^ ions results in positive feedback leading to activation of plasma membrane CRAC channels and sustained T cell activation (137, 138). Ca^2+^ concentration in the mitochondrial matrix also has important effects on the rate of the TCA cycle as three calcium dependent TCA enzymes: 2-oxyglutarate dehydrogenase, NAD^+^-isocitrate dehydrogenase, and pyruvate dehydrogenase are activated by Ca^2+^ ions leading to increased mitochondrial metabolism (139).

### Mitogen-activated protein kinase

The MAPK family of serine/threonine/tyrosine kinases plays a central and pleitropic role in transducing diverse signals from the environment into nuclear transcription factor activation. They are involved in the cellular responses of T cells to inflammatory cytokines, mitogens, insulin, heat shock, and osmotic stress [reviewed in Ref. (140)]. MAPK is required for glucose and glutamine uptake and metabolism in T cells in a CD28 dependent manner and is required for glutaminase activity (89, 141).

### AMP activated protein kinase

AMP activated protein kinase is important in energy homeostasis within the cell and the sensing of hypoxia due to the increase in AMP to ATP ratio under these conditions [reviewed in Ref. (142)]. The binding of AMP or ADP allows the phosphorylation of AMPK, which activated its serine/threonine kinase activity. AMPK phosporylates acetyl-Co-A carboxylase (ACC1) to inhibit its lipogenic activity and ACC2 to promote expression of carnitine palmitoyltransferase (CPT1A), which is the rate limiting factor for the uptake and oxidation of lipids in the mitochondria. PGC1α activity (see above) is also promoted by AMPK activation. AMPK also inhibits TORC1 signaling by phosphorylating TSC1/2 and enhances autophagy, glucose uptake, and mitochondrial biogenesis. The net effect of AMPK activation is to shut down energy intensive processes and to activate pathways that replenish ATP levels within the cell.

## Metabolic Feedback and “Moonlighting” Functions of Metabolic Enzymes

**Figure 5:**
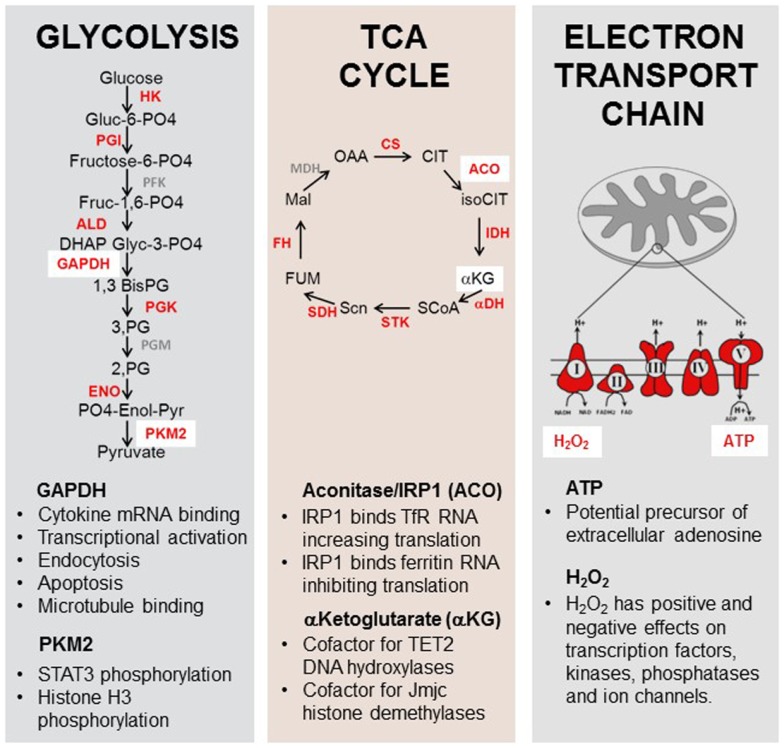
**Moonlighting functions of metabolic enzymes and metabolites**. Enzymes and metabolites of the glycolysis, TCA cycle, and electron transport chain play roles in immune function. Enzymes and substrates in red have been shown to have additional non-metabolic functions in eukaryotes [reviewed in Ref. (173–176)]. GAPDH can bind to the 3′UTR of some cytokine genes, inhibiting their translation. Pyruvate kinase M2 has been shown to have kinase activity for the pro-inflammatory transcription factor STAT3. Aconitase functions as a rheostat for cellular iron in addition to its role in the TCA cycle. α-Ketoglutarate is an essential cofactor for the enzymes TET2 and jumonji-C histone demethylases. ATP may act as a precursor for extracellular adenosine production and H_2_O_2_ has been shown to possess a signaling role in multiple cytoplasmic and nuclear pathways.

In addition to carrying out metabolic activities, many enzymes of the glycolytic, pentose phosphate, TCA, and fatty acid metabolism pathways have dual function and “moonlight” as RNA binding proteins, transcriptionally controlling their targets in a metabolite dependent fashion (Figure 5). This area has been well reviewed (96, 143, 144) so only a few key examples will be highlighted here.

The most thoroughly investigated example of a metabolic enzyme having a second function in RNA translational control is cytosolic aconitase/iron regulatory protein 1 – IRP1 (145, 146). This enzyme functions to convert citrate to isocitrate in the TCA cycle. It is dependent on replete levels of cellular iron for enzymatic function as it contains a cubane 4fe–4S iron sulfur cluster responsible for its catalytic activity. In conditions of low iron this cluster disassembles and converts to IRP1, which is capable of binding to iron responsive elements (IREs), conserved hairpin structures in the 3′ UTR of RNAs responsible for iron homeostasis (147). IRP binds with very high affinity (Kd 5pM) to transferrin receptor RNA (148) to protect the RNA from degradation and increase translation. It simultaneously binds to an IRE in ferritin, an iron storage protein, causing it to become translationally repressed (149). In this way, the enzymatic activity of the protein, dependent on iron senses cellular iron levels and acts as a rheostat for iron by adjusting the translation of RNA encoding proteins involved in iron homeostasis. Uptake of iron is also essential for the heme containing proteins in the electron transport chain, and would be required for resting Treg cells preferentially engaging FAO and OXPHOS.

Several enzymes of the glycolysis pathway have been shown to have RNA binding activity. Glyceraldehyde 3 phosphate dehydrogenase (GAPDH), aldolase, lactate dehydrogenase (LDH), phosphoglycerate kinase (PGK), and glucose 6 phosphate dehydrogenase (G6PDH) have been shown to regulate the translation of immunologically relevant mRNA targets (150–152). GAPDH, LDH PGK, and G6PDH all share a dinucleotide binding region termed the Rossman fold (153). This RNA binding region consists of two βαβ folds each of which binds a mononucleotide. The Rossman fold RNA binding activity is competed for by the dinucleotides NAD^+^, NADH, and ATP. In the case of GAPDH, the cofactor NAD^+^ is required for glycolytic activity and inhibits target RNA binding (150) In this way, when GAPDH is required for glycolysis and cofactors are abundant its RNA binding ability is competed for by cofactors and shut off. GAPDH has been shown to target many RNAs including mRNA (150), tRNA (154), rRNA (155), and viral RNAs (156). GAPDH mRNA targets include IL-2, GM-CSF, IFNα (150), GLUT1 (151), and IFN-γ (95) where it binds to an AU rich region in the 3′ untranslated region. In the case of IFN-γ, GAPDH comprises a component of the gamma interferon-activated inhibitor of translation (GAIT) complex (95). If activated T cells are deprived of glucose, and instead provided with galactose, then glycolysis cannot take place, and yet the T cells still activate and proliferate (because galactose provides alternative precursors for nucleotide synthesis via the pentose phosphate pathway), but now because GAPDH has no substrate, it blocks the translation of IFN-γ. Under these conditions the T cells also then express other markers of T cell exhaustion such as programed death 1 (PD-1) (95). The corollary of this is that inducing glycolysis, for example, by mTOR activation, will tend to promote effector cell differentiation. There are also suggestions that there may be other examples where metabolic enzymes, for example, hexokinase (157) and IDO (51) can have a secondary, signaling role, in DC differentiation.

Pyruvate kinase (PK) catalyzes the final step of glycolyis; phosphoenolpyruvate to pyruvate. It exists in two differentially spliced forms in most cells PKM1 and PKM2 (158). Highly proliferating cells including embryonic cells tumor cells and activated T cells preferentially express the less efficient form PKM2, which may support accumulation of glycolytic intermediates necessary for production of amino acids and nucleotides during proliferation (96, 159, 160). PKM2 also “moonlights” as a kinase for STAT3 using PEP instead of ATP as a phosphate donor (161). This finding suggests that the metabolic status of the T cell, anabolic or catabolic, indirectly controls activity of a transcription factor known to transduce pro-inflammatory signals downstream of diverse cytokines including interferons, IL-5, IL-6, and leptin. Effector T cells in anabolic mode might be predicted to enhance transcriptional programs normally associated with these pro-inflammatory signals.

## Treg Cell Epigenetics and Metabolism

Constitutive expression of FOXP3 has been shown to be essential for continued Treg cell maintenance of functional tolerance *in vivo* (11, 162–164). However, FOXP3 expression on its own does not seem to be sufficient to enable full differentiation into the Treg cell lineage. Mature Treg cells have a characteristic epigenetic “fingerprint” of demethylated genes associated with Treg cell function, which includes five genes termed the “Treg.me”; FOXP3, CTLA4, Ikzf2 (Helios), Ikzf4 (Eos), and Tnfrsf18 (GITR) (165, 166). In addition, several hundred proteins have been found to be associated with FOXP3 in mass spectroscopy screens (167) several of which are important for maintaining FOXP3 transcription. Several important transcription factors including Runx1, NFAT, and GATA3, which are necessary for FOXP3’s function also associate with FOXP3. Importantly, progression along the Treg cell lineage seems to occur prior to the induction of FOXP3 as several characteristic Treg cell gene sets can be observed in T cells from mice with a targeted disruption of the FOXP3 gene into which GFP has been inserted under the control of the FOXP3 promoter. FOXP3 is thought to amplify the pre-existing gene profile (168). Characteristic epigenetic modifications have been shown to be associated with the FOXP3 gene in mature Treg cell, which are not present in naïve T cells. An intronic conserved non-coding element (CNS2) was discovered to be demethylated preferentially in stable Treg cells (169, 170). Histones surrounding this region were also shown to have characteristic modifications of open chromatin (H3K4me3 and acetyl H4 high) (169). DNA methylation and histone modification by methylation, acetylations, and phosphorylation via the action of methyl transferases, acetyl transferases, and kinases, respectively, requires cellular metabolites as enzymatic substrates (171). For example, the sirtuins (histone deacetylases) and poly ADP ribose polymerases (PARPs) require the coenzyme NAD^+^ to function (172). In addition, the TET2 DNA hydroxylases, involved in demethylation of DNA and the jumonji-C (JmjC) histone demethylases involved in histone demethylation belong to a group of enzymes called the α-ketoglutarate dependent enzymes, which require the TCA cycle metabolite α-ketoglutarate to function in addition to ascorbate, oxygen, and iron. Both these classes of enzymes are inhibited by the TCA cycle intermediates fumarate and succinate. It is conceivable that epigenetic changes in Treg cells required for stability and reprograming to the Treg cell lineage may be influenced by the metabolic program that the cell adopts in response to environmental stimuli such as glucose or fatty acid availability.

## Are Regulatory T Cells Adapted Metabolically for Current and Future Microenvironments?

The experimental data of Treg cell metabolism are predominantly derived from *in vitro* observations of resting Treg cells. These cell cultures are usually performed in media with a vast molar excess of EAAs, 10-fold physiological levels of glucose and glutamine and oxygen concentrations in excess of physiological norms. However, it is well known that Treg cells proliferate vigorously *in vivo* and presumably require glycolytic intermediates to fuel anabolic demand during multiple rounds of mitosis. The question remains, what would be the advantage to non-proliferating Treg cells of adopting FAO and OXPHOS as a default metabolic mode? We hypothesize that Treg cells are uniquely adapted to their current and future *in vivo* environments. While Treg cells interact with DCs in a tolerant microenvironment they induce EAA-catabolizing enzyme expression in the DC, forming a zone of acute EAA starvation. In this situation their catabolic mode preferentially protects them from the effects of amino acid starvation, and presumably limiting glucose concentrations in inflamed environments. In addition, they receive survival signals in the form of IL-2 from effector T cells, yet produce little IL-2 themselves, thus, inhibiting further effector T cell proliferation. It is possible that shifting metabolism to catabolic mode in this situation frees up “moonlighting” glycolytic enzymes to suppress translation of pro-inflammatory cytokines by the Treg cells. Expansion of Treg cell numbers in the draining lymph nodes of inflammatory sites would elicit a switch to anabolic metabolism under conditions of sufficient glucose and glutamine, enabling increase in cell mass and mitosis. The ability to shift from an anabolic expansion mode to a catabolic suppression mode may be key to Treg cell function, and presents an attractive target for therapeutic intervention.

## Concluding Remarks

Nutrient sensing and the coordination of metabolism seem to be inherently associated with the mechanisms of immune regulation *in vivo*. The question that then arises is – can any of these metabolic processes be specifically targeted for manipulating immune responses in transplantation, the treatment of autoimmune diseases and cancer immunotherapy? Many of these pathways are common to many different cells in the body and relying on the immunosuppressive effects of available drugs such as the mTOR inhibitors may therefore have a variety of unwanted side effects. Consequently, we need to look either for potential target components of these metabolic pathways that are restricted primarily to immune cells or for ways to amplify the effects of metabolic inhibitors such that they can be used at doses well below that which have effects outside the immune system. One way to achieve this might be to concentrate on the period of immune reconstitution after lymphocyte depletion when the metabolic needs of homeostatic proliferation of a small number of residual T cells could be biased in favor of regulatory T cells.

## Conflict of Interest Statement

The authors declare that the research was conducted in the absence of any commercial or financial relationships that could be construed as a potential conflict of interest.
